# Systems Barriers to Medication Management During Hospital to Home Transitions of Older Adults With Dementia

**DOI:** 10.1093/geroni/igab046.876

**Published:** 2021-12-17

**Authors:** Maningbe Keita-Fakeye, Rhea Sharma, Sylvan Greyson, Quincy Samus, Ayse Gurses, Sara Keller, Alicia Arbaje

**Affiliations:** 1 Johns Hopkins University, Parkville, Maryland, United States; 2 Virginia Commonwealth University, Virginia Commonwealth University, Virginia, United States; 3 Bayview Medical Center, Baltimore, Maryland, United States; 4 Johns Hopkins University School of Medicine, Baltimore, Maryland, United States; 5 Johns Hopkins School of Medicine, Baltimore, Maryland, United States; 6 Johns Hopkins School of Medicine, Lutherville-Timonium, Maryland, United States

## Abstract

The hospital-to-home transition is a high-risk period for medication errors and adverse events for older adults living with dementia. Researchers conducted a qualitative study using semi-structured interviews and participant solicited diaries. Caregivers of adults ages 55 and older were recruited to understand barriers to medication management during hospital to skilled home health care transitions. We used a human factors engineering approach to guide our understanding of systems level barriers. At least two researchers independently coded each transcript using content analysis and the ATLAS.ti software. We interviewed 23 caregivers and identified five barrier types stemming from systems breakdowns related to: (1) knowledge and information, (2) access to and use of resources and tools, (3) caregiver burden, (4) pandemic concerns, and (5) health limitations. Caregivers grappled with receiving overwhelming, insufficient, incorrect, or conflicting information, and had difficulty managing information from different sources. Latinx caregivers encountered language barriers that impeded role and task clarity. Caregivers expressed mistrust in health systems elements and inability to access resources. Caregivers were in need of additional caregiving assistance, financial aid, and tools to manage medications. Balancing multiple medications and responsibilities left caregivers burdened. The health limitations of the older adult and COVID-19 concerns related to reduced access to resources and ability to deliver and receive in person care complicated task management. Altogether these barriers reflect systems level breakdowns impeding task understanding, execution, and overall management. These findings will inform the development of interdisciplinary strategies to ensure safer care transitions.

